# Evaluation of Efficacy and Safety in First‐Line Treatment Methods for Extensive‐Stage Small Cell Lung Cancer: A Comprehensive Comparative Study of Chemotherapy, Targeted Therapy Combined With Chemotherapy, and Immunotherapy Combined With Chemotherapy

**DOI:** 10.1111/crj.13819

**Published:** 2024-08-08

**Authors:** Tiantian Zhang, Lu Tao, Yufo Chen, Shanshan Zhang, Yang Liu, Yumei Li, Rui Wang

**Affiliations:** ^1^ Departments of Medical Oncology The First Affiliated Hospital of Bengbu Medical College Bengbu Anhui People's Republic of China; ^2^ Anhui Provincial Key Laboratory of Cancer Translational Medicine The First Affiliated Hospital of Bengbu Medical College Bengbu Anhui People's Republic of China

**Keywords:** antiangiogenic therapy, efficacy, immunotherapy, safety, small cell lung cancer

## Abstract

**Background:**

Small cell lung cancer (SCLC) is a highly aggressive tumor with limited effectiveness in its standard chemotherapy treatment. Targeted antiangiogenic therapy and immune checkpoint inhibitors (ICIs) have demonstrated potential as alternative treatments for extensive‐stage SCLC (ES‐SCLC). However, there is insufficient comparative evidence available to determine the optimal first‐line treatment option between ICIs plus chemotherapy and targeted antiangiogenic therapy plus chemotherapy.

**Objective:**

This study is aimed at analyzing clinical data from ES‐SCLC patients treated at the First Affiliated Hospital of Bengbu Medical College between June 2021 and June 2023. The study compared the efficacy and safety of three first‐line treatment regimens: standard chemotherapy, antiangiogenic therapy combined with chemotherapy, and immune combination therapy.

**Methods:**

Patients who met the inclusion criteria were divided into three groups: chemotherapy, immune combination therapy, and antiangiogenic therapy combined with chemotherapy. The study collected data on clinical characteristics, treatment regimens, and adverse reactions. The analysis included objective response rate (ORR), duration of response (DoR), disease control rate (DCR), progression‐free survival (PFS), and treatment safety.

**Results:**

A total of 101 patients were included in the study, with 49 receiving chemotherapy alone, 19 receiving antiangiogenic therapy, and 33 receiving immune combination therapy. The ORRs were 78.9% for antiangiogenic therapy, 72.7% for immune combination therapy, and 42.9% for chemotherapy alone. The median PFS was 8.0 months for antiangiogenic therapy, 7.8 months for immune combination therapy, and 5.2 months for chemotherapy alone. Both combination therapy groups demonstrated superior efficacy compared to chemotherapy alone.

**Conclusion:**

Targeted combined chemotherapy and immune combination chemotherapy showed superior efficacy as first‐line treatments for ES‐SCLC compared to chemotherapy alone, with manageable adverse reactions.

## Graphical abstract

Evaluating efficacy and safety of first‐line treatments for extensive‐stage small cell lung cancer: chemotherapy, targeted therapy + chemotherapy, and immunotherapy + chemotherapy. We compared the clinical data of 101 patients receiving standard chemotherapy, antiangiogenic therapy + chemotherapy, or immune combination therapy. The combination therapy groups showed higher objective response rates, longer progression‐free survival, and manageable adverse reactions compared to chemotherapy alone. Our findings support the use of targeted combined chemotherapy and immune combined chemotherapy as first‐line treatments for ES‐SCLC.

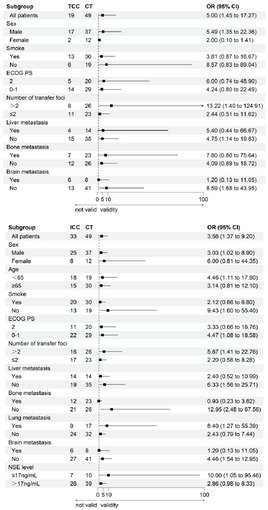

## 1 Introduction

Small cell lung cancer (SCLC) is a highly invasive neuroendocrine carcinoma, representing approximately 15% of all lung cancer cases [1]. The 2021 National Comprehensive Cancer Network (NCCN) guidelines introduced a novel staging method for SCLC, incorporating the Veterans Administration Lung Study Group (VALG) and American Joint Committee on Cancer (AJCC) systems. This approach, utilizing VALG for disease extent determination and AJCC's revised TNM staging for lung cancer, enables a more precise SCLC staging definition, significantly impacting therapeutic strategies. Patients categorized as limited‐stage SCLC (LS‐SCLC), defined as AJCC Stages I–III (any T, any N, and M0), are eligible for radiotherapy (RT), with surgical resection considered for select T1–T2, N0 stage patients. Extensive‐stage SCLC (ES‐SCLC) corresponds to AJCC Stage IV (any T, any N, and M1a/b/c) or T3–T4 due to multiple lung nodules. The recommended first‐line treatment for ES‐SCLC is the EP regimen, combining chemotherapy with etoposide or irinotecan and platinum drugs, with carboplatin as a viable alternative for cisplatin‐intolerant patients. This staging and grading approach offers clear therapeutic guidance and holds significant relevance for enhancing patient clinical outcomes. TNM staging evaluates tumor size (T), lymph node status (N), and distant metastasis presence (M), with T1–T2 referring to tumors localized to one lung ≤ 5 cm in diameter, N0 indicating no lymph node metastases, and M0 signifying no distant metastases. Initially, about 70% of SCLC patients are diagnosed with extensive‐stage disease (ES‐SCLC), which is associated with a poor prognosis. The median overall survival (OS) is only 10 months [2], and the 5‐year survival rate is a dismal 7% [3]. Despite the use of standard platinum/etoposide chemotherapy as the first‐line treatment, the majority of patients experience disease progression or relapse within a few months [4]. Unfortunately, there is currently a lack of more effective treatment options for recurrent or drug‐resistant cases. Several alternative treatment approaches have been explored over the years; however, their outcomes remain limited.

The emergence of antiangiogenic therapy and immunotherapy has opened up new possibilities for the treatment of SCLC. In particular, immune checkpoint inhibitors (ICIs), such as PD‐L1 monoclonal antibodies, have shown improved prognostic effects in patients with ES‐SCLC. The IMpower133 [5] and CASPIAN trials [6] have confirmed the significant enhancements in OS and progression‐free survival (PFS) achieved through the combined use of chemotherapy and ICIs. However, the high cost of ICIs limits their clinical application in ES‐SCLC patients [7], making domestically developed PD‐1 drugs a potential alternative. Tislelizumab, a highly specific and affinity‐enhanced PD‐1 inhibitor, enhances antitumor clearance by reducing binding to macrophages and boosting T‐cell response [8]. Compared to other PD‐1 inhibitors, tislelizumab has higher affinity and lower off‐target rates [9]. A Phase II study revealed that combining tislelizumab with platinum‐etoposide therapy demonstrated promising antitumor efficacy in ES‐SCLC, with a median PFS (mPFS) of 6.9 months and a 1‐year survival rate of 76% [10]. However, further research and comparisons are needed to validate these findings.

In addition to immunotherapy, targeted antiangiogenic drugs like anlotinib have shown potential in improving the prognosis of SCLC patients. Anlotinib, an orally administered small‐molecule receptor tyrosine kinase inhibitor (TKI), inhibits tumor angiogenesis and proliferation by targeting key receptors [11]. The ALTER 1202 study demonstrated that anlotinib, as a third‐line treatment for ES‐SCLC, can prolong both PFS and OS, while increasing the disease control rate (DCR) [12]. Furthermore, preliminary results have indicated the potential of combining anlotinib with chemotherapy as a first‐line treatment for ES‐SCLC. Initial findings from a Phase II clinical study evaluating the efficacy and safety of anlotinib in combination with EP/EC chemotherapy for first‐line treatment of ES‐SCLC were presented at the 2021 American Society of Clinical Oncology (ASCO) meeting. The trial, which began in August 2020, included 20 patients for efficacy evaluation. The results showed an mPFS of 10.0 months, a median OS of 15.0 months, an objective response rate (ORR) of 90%, and a DCR of 100% [13]. These results highlight the promising potential of combining targeted antiangiogenic therapy with chemotherapy as a first‐line treatment strategy for ES‐SCLC.

However, there is still a lack of high‐level evidence from randomized controlled trials to support the combination of tislelizumab and chemotherapy, as well as anlotinib and chemotherapy, as first‐line treatment strategies for ES‐SCLC. Therefore, this article aims to conduct a clinical retrospective study to analyze the efficacy and safety of standard chemotherapy, tislelizumab combined with chemotherapy, and anlotinib combined with chemotherapy as first‐line treatment options for patients with ES‐SCLC. The goal of this study is to provide more definitive evidence‐based support for clinical practice.

## 2 Methods

### 2.1 Study Design and Patients

This study is a retrospective, real‐world research conducted at a single center without intervention. The study cohort consists of patients with ES‐SCLC who received first‐line treatment with EP/C (CT cohort), anlotinib combined with EP/C (TCC cohort), or tislelizumab combined with EP/C (ICC cohort) between June 1, 2021, and June 30, 2023, at our institution. Clinical characteristics of the patients, such as age, gender, smoking status, ECOG performance score, number and location of metastatic lesions, and treatment‐related adverse reactions, were collected.

### 2.2 Inclusion and Exclusion Criteria

The inclusion criteria for patient selection were as follows: (1) age ≥ 18 years; (2) patients diagnosed with ES‐SCLC confirmed by pathology, with measurable lesions according to the Response Evaluation Criteria in Solid Tumors (RECIST) Version 1.1; (3) ECOG performance status ≤ 2; (4) normal functioning of major organs; (5) availability of diagnostic imaging, including baseline and follow‐up contrast‐enhanced computed tomography (CT) scans of the thorax and abdomen, performed using a standardized protocol to assess disease extent and response to treatment; and (6) willingness and capability to undergo 18F‐fluorodeoxyglucose positron emission tomography (18F‐FDG PET) scans as part of the standard staging and restaging procedures, where indicated for the evaluation of metabolic activity and detection of distant metastases.

The exclusion criteria for patient selection were as follows: (1) severe lack of clinical records or loss of follow‐up, (2) inability to conduct imaging efficacy evaluation, and (3) patients with active bleeding or serious systemic diseases.

### 2.3 Therapeutic Methods

- Chemotherapy group (CT): etoposide (100 mg/m^2^, intravenous infusion, on Days 1, 2, and 3) in combination with a platinum‐based agent (cisplatin: 25 mg/m^2^, intravenous infusion, on Days 1–4; or carboplatin: target area under the curve [AUC] of 4–5, intravenous infusion, on Day 1). Each cycle lasts for 21 days. Treatment continues until disease progression or intolerable toxic reactions.
- Anlotinib in combination with chemotherapy group (TCC): anlotinib (12 mg, oral administration, from Day 1 to Day 14) in combination with EP/C (same chemotherapy regimen as the CT group). Each cycle lasts for 21 days. After receiving 4–6 cycles of treatment, anlotinib monotherapy is continued until disease progression or intolerable toxic reactions.
- Tislelizumab in combination with chemotherapy group (ICC): tislelizumab (200 mg, intravenous infusion, on Day 1) in combination with EP/C (same chemotherapy regimen as the CT group). Each cycle lasts for 21 days. After receiving 4–6 cycles of treatment, tislelizumab monotherapy is continued until disease progression or intolerable toxic reactions.

### 2.4 Efficacy and Safety Evaluation

This study employed a retrospective design. The assessment of target lesions was conducted every 2–3 cycles or as clinically necessary, adhering to RECIST Version 1.1. The primary goal was the ORR, which indicates the proportion of confirmed complete response (CR) and partial response (PR). Secondary goals included PFS, defined as the duration from enrollment to progression disease (PD) or death from any cause, whichever came first. Other secondary goals encompassed DCR, covering patients with CR, PR, and stable disease (SD), as well as the duration of response (DoR), which is the period from confirmed CR or PR to disease progression or death due to any cause. CT scans were used to evaluate chest target lesions, while target lesions in other locations were assessed using either CT or MRI scans. The incidence and severity of adverse events (AEs) were monitored according to the National Cancer Institute Common Terminology Criteria for Adverse Events Version 4.03. Changes in clinical laboratory values, vital signs, and physical examinations were also evaluated.

### 2.5 Statistical Analysis

Data analysis and graphical representation were performed using GraphPad Prism 6 software (GraphPad Software, San Diego, United States) and RStudio 8.14.179693 software. Categorical variables were assessed using the chi‐square test, while continuous variables were analyzed using the Mann–Whitney *U* test. Kaplan–Meier survival curves were generated to compare survival differences among the three treatment groups, and the corresponding median survival times for each population were calculated. A Cox proportional hazards regression analysis was conducted to evaluate potential biomarkers or baseline characteristics through univariate and multivariate analyses, estimating hazard ratios (HRs) and 95% confidence intervals (CIs).

## 3 Results

### 3.1 Patient Selection Diagram

**FIGURE 1:**
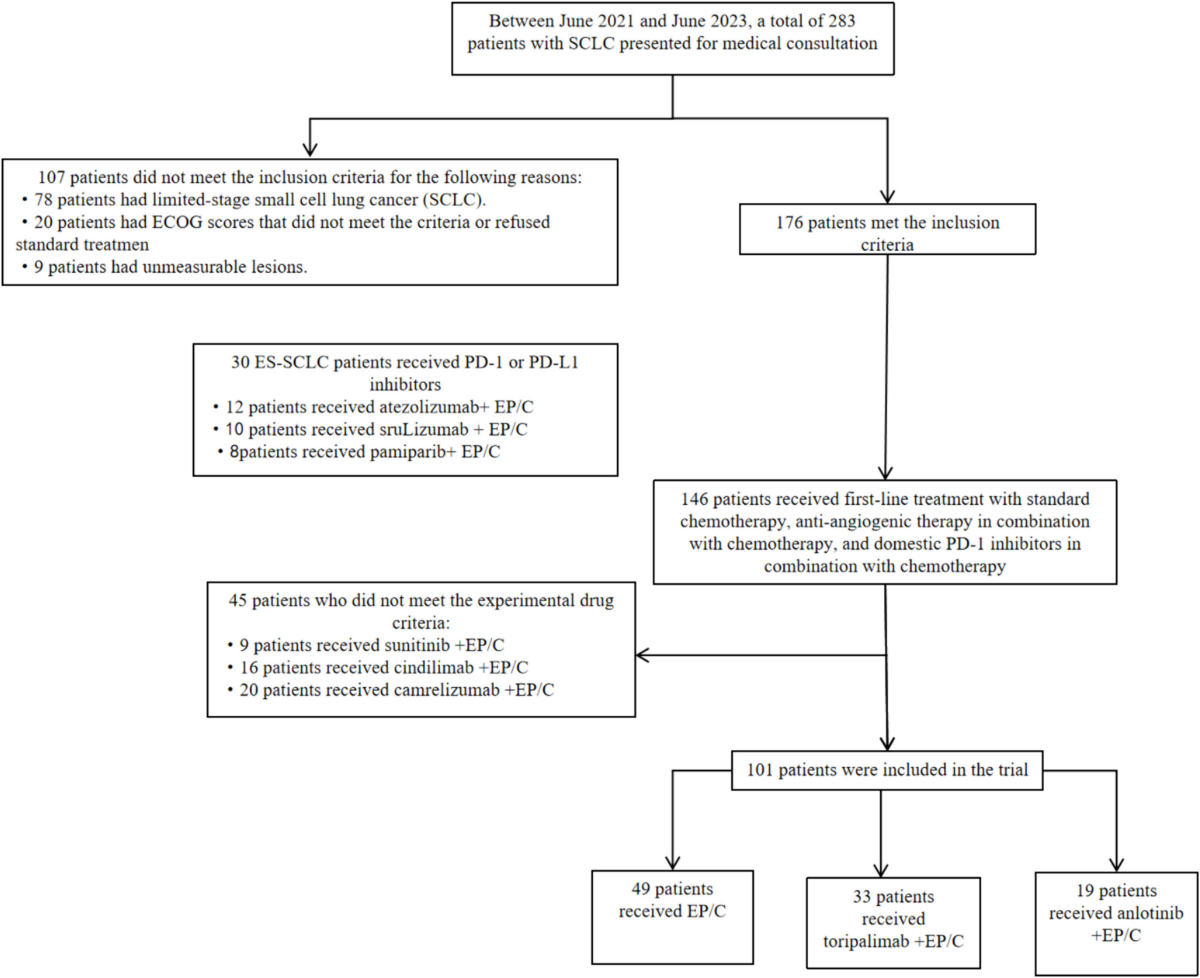
Diagram presenting the screening process of patients with extensive‐stage small cell lung cancer (SCLC) who underwent first‐line chemotherapy, immunotherapy in combination with chemotherapy, and targeted therapy in combination with chemotherapy between June 2021 and June 2023.

Between June 2021 and June 2023, a total of 283 individuals diagnosed with SCLC sought treatment at the Department of Oncology, First Affiliated Hospital of Bengbu Medical College. Among them, 78 were diagnosed with LS‐SCLC, while 205 received an ES‐SCLC diagnosis. Eventually, 101 patients with ES‐SCLC were included in the study. This group consisted of 49 individuals who received EP/C chemotherapy, 33 who received tislelizumab combined with EP/C chemotherapy, and 19 patients who received anlotinib combined with EP/C chemotherapy. The selection process is visually presented in Figure 1.

### 3.2 Baseline Clinical Characteristics of Patients

The baseline clinical characteristics of the 101 patients are presented in Table 1. Among them, 79 cases (78.2%) were male, while 22 cases (21.8%) were female. The median age was 67 years (range: 37–82 years), with 58 cases (57.4%) being 65 years or older and 43 cases (42.6%) being younger than 65 years. There were no statistically significant differences observed among the three groups in terms of gender, age, smoking history, alcohol consumption history, ECOG PS, site and number of metastases, and baseline NSE levels (*p* > 0.05).

**TABLE 1:** Baseline clinical characteristics of patients.

	CT (*n* = 49)	TCC (*n* = 19)	ICC (*n* = 33)	*p*	*χ* ^2^
Median age range	67 (50–82)	67 (51–81)	62 (37–81)		
Years, *n* (%)				0.444	1.624
< 65	19 (38.8%)	7 (36.8%)	17 (51.5%)		
≥ 65	30 (61.2%)	12 (63.2%)	16 (48.5%)		
Sex, *n* (%)				0.419	1.741
Female	12 (24.5%)	2 (10.5%)	8 (24.2%)		
Male	37 (75.5%)	17 (89.5%)	25 (75.8%)		
Smoking status				0.832	0.368
Yes	30 (61.2%)	13 (68.4%)	20 (60.6%)		
No	19 (38.8%)	6 (31.6%)	13 (39.4%)		
ECOG status				0.504	1.369
0–1	29 (59.2%)	14 (73.7%)	22 (66.7%)		
2	20 (40.8%)	5 (26.3%)	11 (33.3%)		
Number of transfer foci				0.713	0.678
≤ 2	23 (46.9%)	11 (57.9%)	17 (51.5%)		
> 2	26 (53.1%)	8 (42.1%)	16 (48.5%)		
Lung metastases				0.295	2.44
Yes	17 (34.7%)	3 (15.8%)	9 (27.3%)		
No	32 (65.3%)	16 (84.2%)	24 (72.7%)		
Liver metastases				0.226	2.97
Yes	14 (28.6%)	4 (21.1%)	14 (42.4%)		
No	35 (71.4%)	15 (78.9%)	19 (57.6%)		
Bone metastases				0.57	1.124
Yes	23 (46.9%)	7 (36.8%)	12 (36.4%)		
No	26 (53.1%)	12 (63.2%)	21 (63.6%)		
Brain metastases				0.352	2.087
Yes	8 (16.3%)	6 (31.6%)	6 (18.2%)		
No	41 (83.7%)	13 (68.4%)	27 (81.8%)		
Pleura metastases				0.186	3.366
Yes	14 (28.6%)	7 (36.8%)	5 (15.2%)		
No	35 (71.4%)	12 (63.2%)	28 (84.8%)		
Paranephros metastases				0.652	0.856
Yes	6 (12.2%)	2 (10.5%)	2 (6.1%)		
No	43 (87.8%)	17 (89.5%)	31 (93.9%)		
NSE baseline level				0.885	0.245
≤17 ng/mL	10 (20.4%)	3 (15.8%)	7 (21.2%)		
> 17 ng/mL	39 (79.6%)	16 (84.2%)	26 (78.8%)		

Abbreviations: CT, etoposide plus cisplatin or etoposide plus carboplatin; ECOG PS, Eastern Cooperative Oncology Group performance status; ICC, tislelizumab in combination with etoposide plus cisplatin or etoposide plus carboplatin; NSE, neuron‐specific enolase; TCC, anlotinib in combination with etoposide plus cisplatin or etoposide plus carboplatin.

### 3.3 Efficacy

**FIGURE 2:**
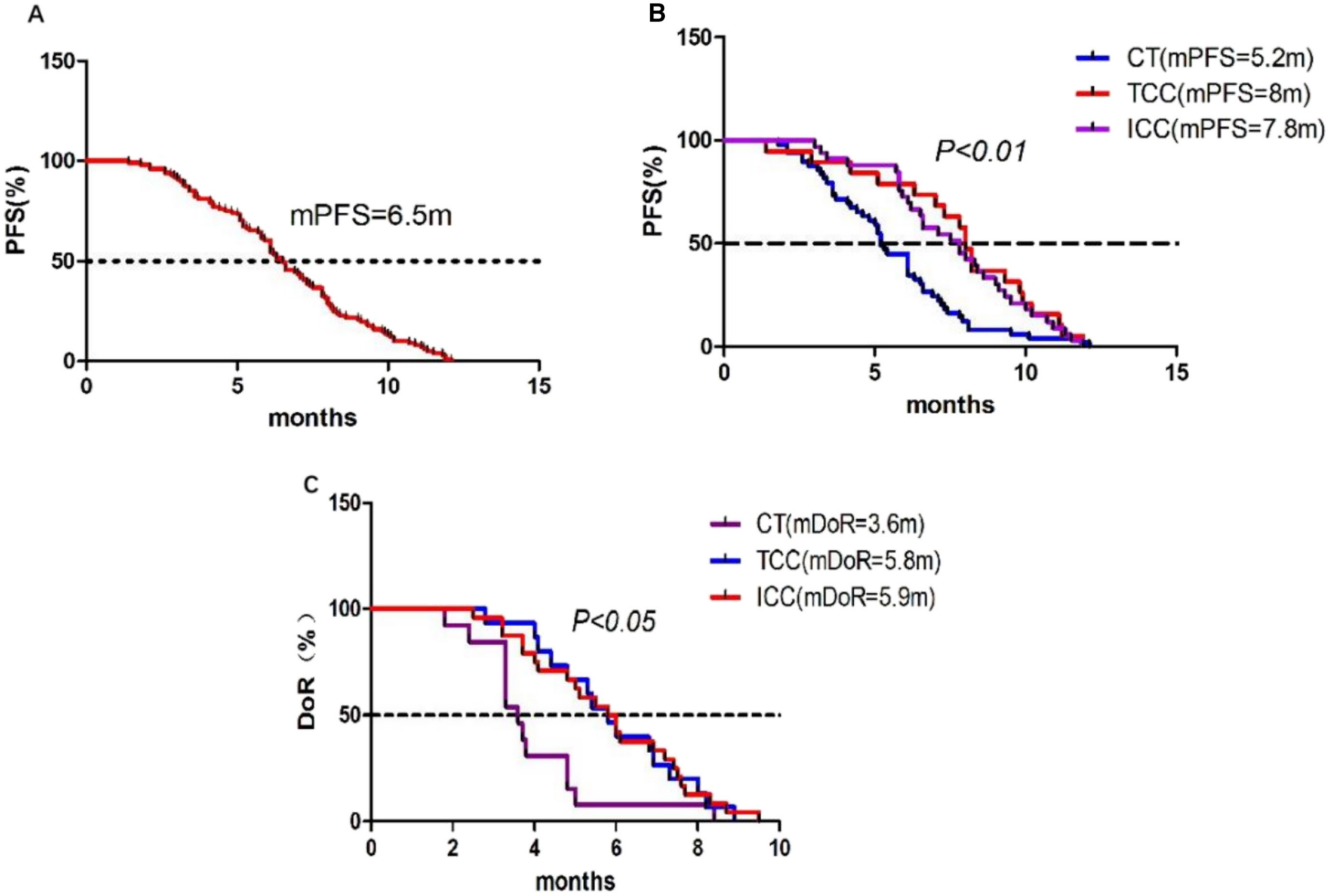
Kaplan–Meier survival curves. (A) The curve represents the progression‐free survival (PFS) in the entire patient cohort. (B) This graph compares the PFS among the three groups. (C) This graph compares the duration of response (DoR) among the three groups.

As of June 30, 2023, all 101 patients were included in the efficacy evaluation cohort. The clinical responses to treatment observed across all study groups are detailed in Table 2. Across the patient cohort, 60 patients (59.4%) achieved a PR, 28 (27.7%) had SD, and 13 (12.9%) experienced progression disease (PD). The confirmed ORR and DCR for the entire cohort were 59.4% and 87.1%, respectively. Examining individual subgroups, the CT subgroup exhibited a confirmed ORR of 42.9%, DCR of 83.7%, and median DoR (mDoR) of 3.6 months. In the TCC subgroup, the confirmed ORR was 78.9%, DCR was 89.5%, and mDoR was 5.8 months. For the ICC subgroup, the confirmed ORR was 72.7%, DCR was 90.9%, and mDoR was 5.9 months. Considering the entire patient cohort, the mPFS was marked at 6.5 months (Figure 2A). The CT subgroup had an mPFS of 5.2 months, the TCC subgroup's mPFS was 8 months, and the ICC subgroup's mPFS was 7.8 months. The differences between these subgroups were statistically significant (Figure 2B). In terms of DoR, the CT subgroup had an mDoR of 3.6 months, the TCC subgroup reported an mDoR of 5.8 months, and the ICC subgroup's mDoR was 5.9 months. These differences were statistically significant (Figure 2C).

**TABLE 2:** Treatment efficacy.

BOR, *n* (%)	CT (*n* = 49)	TCC (*n* = 19)	ICC (*n* = 33)	*p*	*χ* ^2^
CR, *n* (%)	0	0	0		
PR, *n* (%)	21 (42.9%)	15 (79.0%)	24 (72.7%)	0.004	11.002
SD, *n* (%)	20 (40.8%)	2 (10.5%)	6 (18.2%)	0.014	8.496
PD, *n* (%)	8 (16.3%)	2 (10.5%)	3 (9.1%)	0.596	1.035
ORR, *n* (%)	21 (42.9%)	15 (79.0%)	24 (72.7%)	0.004	11.002
DCR, *n* (%)	41 (83.7%)	17 (89.5%)	30 (90.9%)	0.596	1.035
Relieve situation					
Yes	21 (42.9%)	15 (78.9%)	24 (72.7%)	0.004	11.002
No	28 (57.1%)	4 (21.1%)	9 (27.3%)		

Abbreviations: CT, etoposide plus cisplatin or etoposide plus carboplatin; DCR, disease control rate; DoR, duration of response; ICC, tislelizumab in combination with etoposide plus cisplatin or etoposide plus carboplatin; ORR, objective response rate; PD, progression disease; PFS, progression‐free survival; PR, partial response; SD, stable disease; TCC, anlotinib in combination with etoposide plus cisplatin or etoposide plus carboplatin.

### 3.4 Relationship Between Baseline Characteristics and PFS/ORR

**FIGURE 3:**
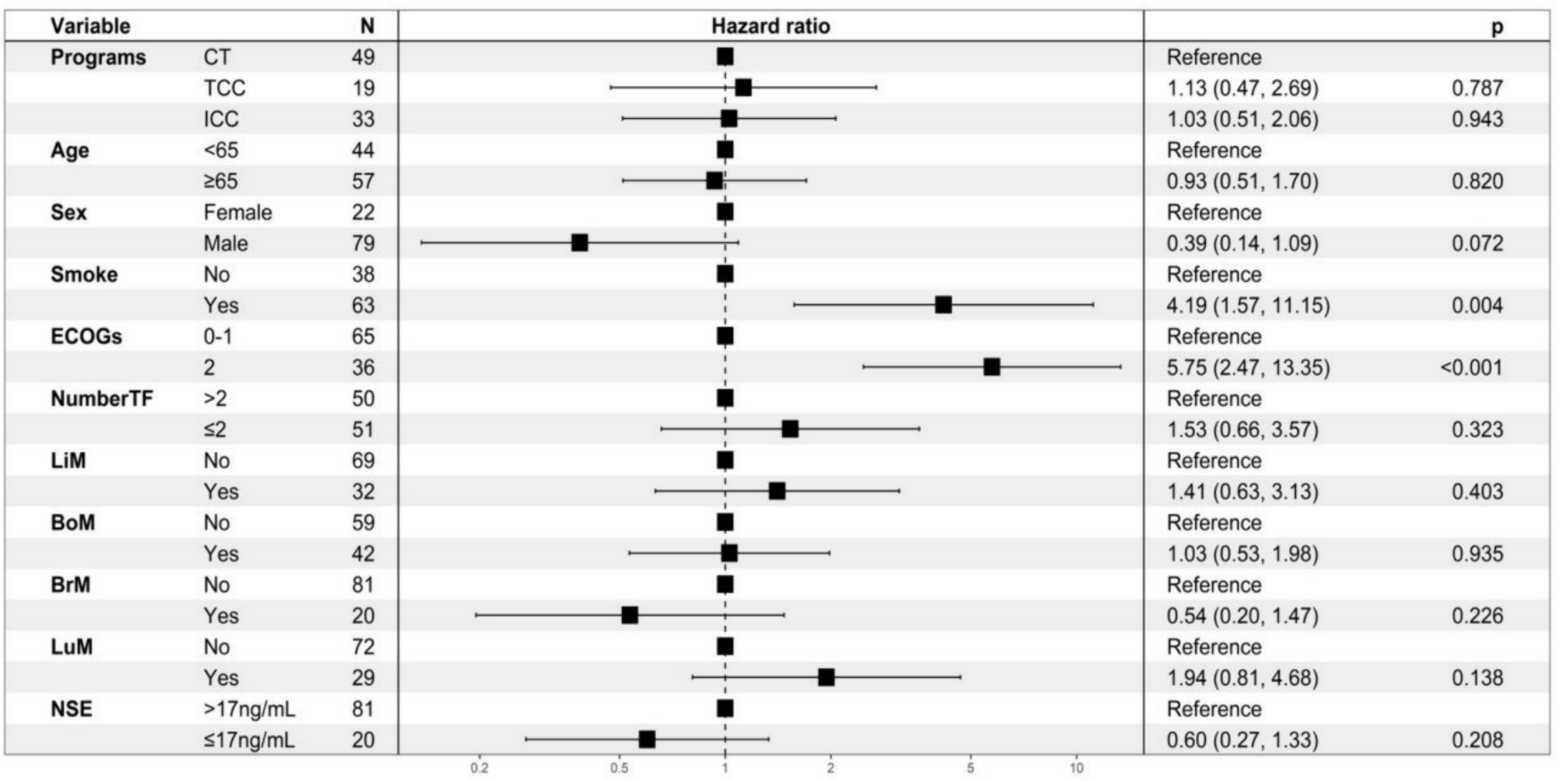
Multivariate Cox regression analysis of PFS‐related factors in all patients.

**FIGURE 4:**
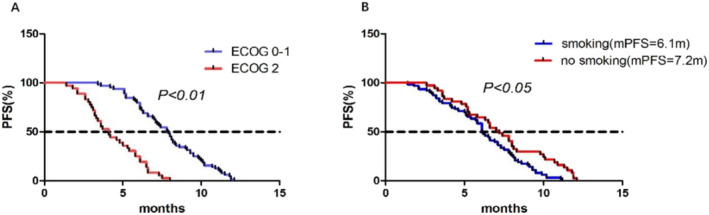
Kaplan–Meier survival curves. (A) The curve compares the survival rates between patients with an ECOG score of 0–1 and ECOG score of 2. (B) The curve compares the survival rates between smokers and nonsmokers.

To identify potential factors associated with PFS, we conducted univariate analyses on all factors and included statistically significant factors (*p* < 0.05) and baseline imbalances in a multivariate Cox regression analysis. The results of the multivariate Cox regression analysis demonstrated that smoking status (no vs. yes: HR = 4.19, 95% CI 1.57–11.15, *p* = 0.004) and ECOG PS (2 vs. 0–1: HR = 0.17, 95% CI 0.07–0.5, *p* < 0.001) were independent factors influencing PFS (Figure 3). Patients with an ECOG score of 0–1 had an mPFS of 7.8 months, while patients with a score of 2 had an mPFS of 4.1 months (Figure 4A). Smokers had an mPFS of 6.1 months compared to 7.2 months for nonsmokers, and both differences were statistically significant (Figure 4B).

**FIGURE 5:**
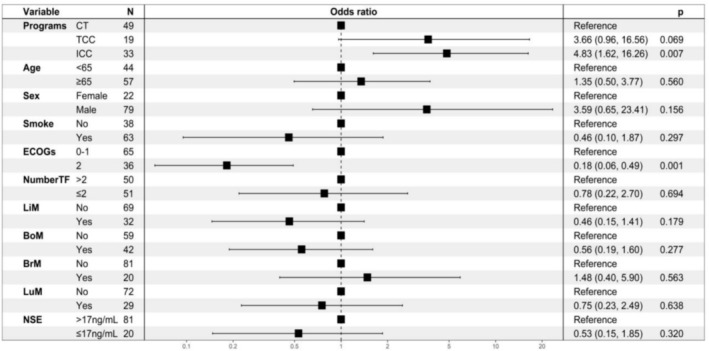
Multivariate Cox regression analysis of ORR‐related factors in all patients.

To determine factors associated with the ORR, we conducted a single‐factor analysis of the ORR based on baseline characteristic subgroups. Statistically significant factors (*p* < 0.05) and baseline imbalances were included in a multivariate Cox regression analysis. The results showed that treatment method and ECOG performance status were independent prognostic factors for ORR. The ORR for patients with an ECOG score of 0–1 was 78.8%, while for those with a score of 2, it was 33.3% (*p* < 0.001). Compared to chemotherapy, immunotherapy combined with chemotherapy significantly improved ORR (*p* = 0.007), and targeted therapy combined with chemotherapy showed a trend towards increased ORR, although the difference was not statistically significant (*p* = 0.069) (Figure 5).

### 3.5 Single‐Factor Subgroup Analysis

**FIGURE 6:**
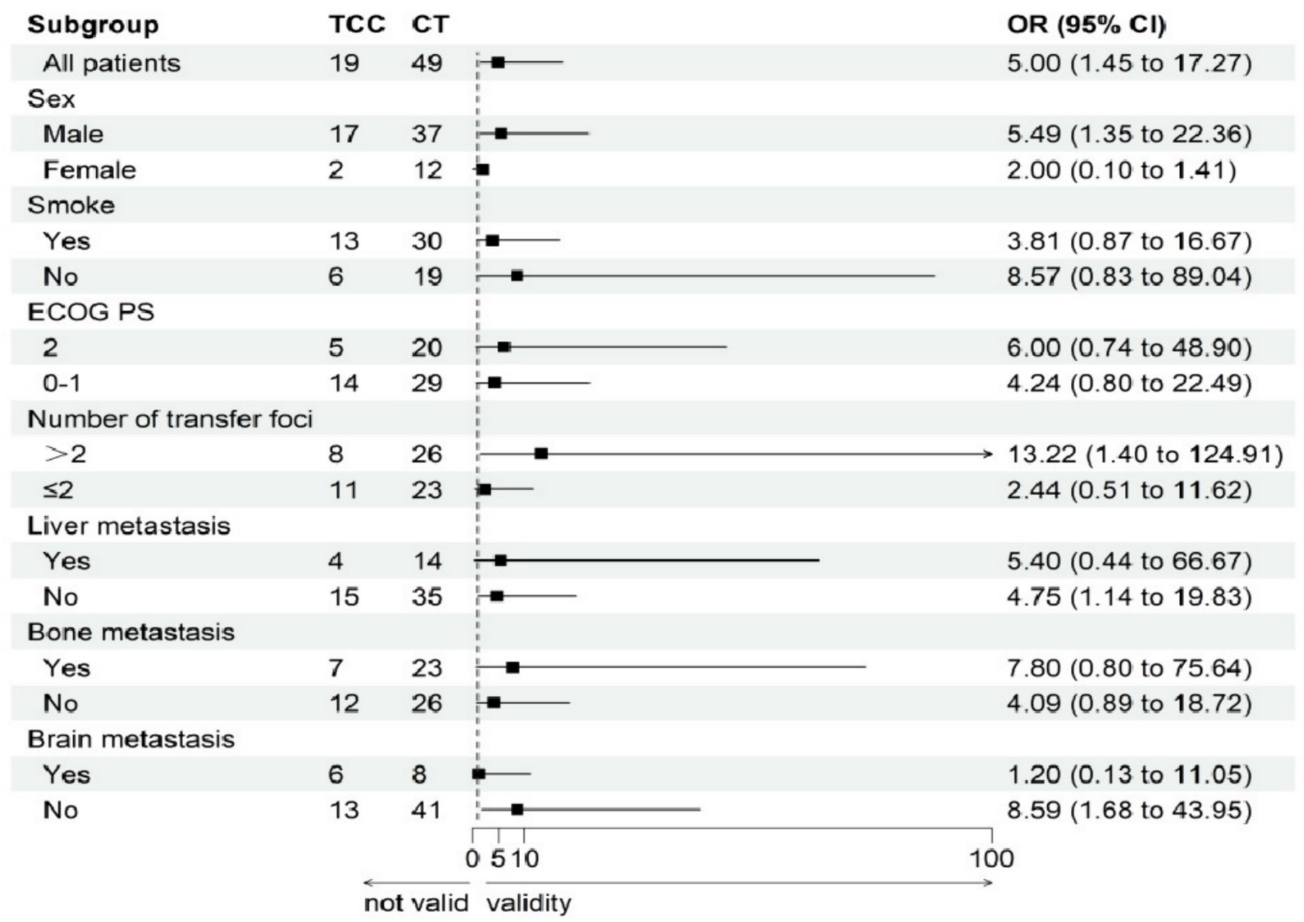
Subgroup analysis of ORR between the targeted therapy and chemotherapy groups.

A single‐factor subgroup analysis was conducted on various factors between the chemotherapy group and the targeted therapy group (Figure 6). Logistic regression subgroup analysis revealed that in the male population, for those with more than two metastatic sites and those without liver or brain metastasis, the combined targeted therapy and chemotherapy group outperformed the chemotherapy group (CI greater than one).

**FIGURE 7:**
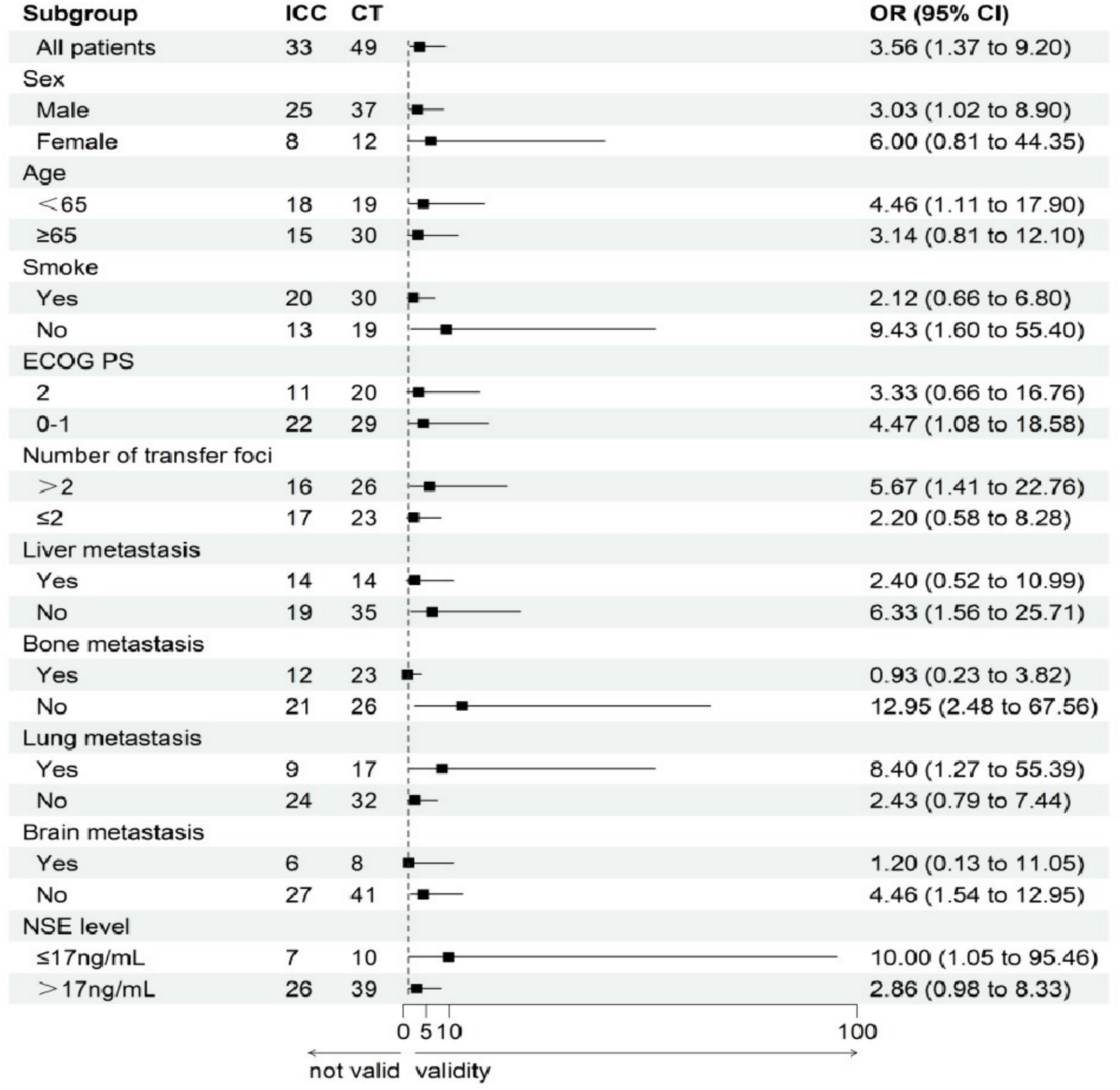
Subgroup analysis of ORR between the immunotherapy and chemotherapy groups.

Similarly, a single‐factor subgroup analysis was performed on various factors between the chemotherapy group and the immunotherapy group (Figure 7). Logistic regression subgroup analysis indicated that in the male population, those below 65 years of age; nonsmokers; with ECOG performance status of 0–1; having more than two metastatic sites; no liver, bone, or brain metastasis; with pulmonary metastasis; and baseline NSE levels below 12 ng/mL, the immunotherapy treatment had better efficacy compared to chemotherapy (CI greater than one).

**FIGURE 8:**
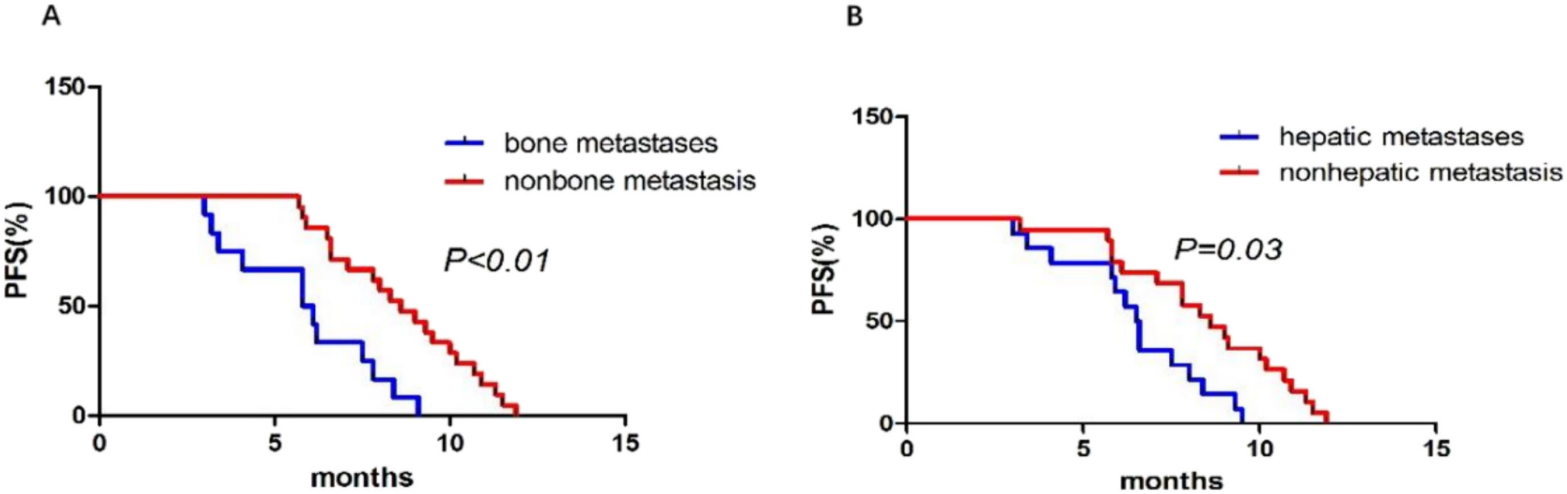
Kaplan–Meier survival curves of different subgroups in immunotherapy group.

Moreover, within the immunotherapy group, patients without bone metastasis showed superior efficacy compared to those with bone metastasis (Figure 8A), with a statistically significant difference. Similarly, patients without liver metastasis also demonstrated better efficacy compared to those with liver metastasis (Figure 8B), with a statistically significant difference.

### 3.6 Safety

As of June 30, 2023, out of 101 patients, 99 (99/101) experienced at least one treatment‐emergent AE (TEAE) related to any study drug. The most common drug‐related adverse reactions were nausea (66), anemia (64), leukopenia (53), and fatigue (52). In the immunotherapy combination group, three patients (9.1%) experienced AE considered by investigators to be related to tislelizumab, including one case of immune‐related pneumonia and two cases of immune‐related hepatitis. No Grade 3 or higher immune‐related AEs occurred. In the targeted therapy combination group, nine patients (47.4%) experienced AE considered by investigators to be related to anlotinib. The most common anlotinib‐related AEs were hypertension (55.6%, *n* = 5) and hand–foot skin reaction (44.4%, *n* = 4). Forty‐three patients (79.6%) experienced TEAEs of Grade ≥ 3 related to any study drug. The most common Grade ≥3 TEAEs related to any study drug were neutropenia (48.1%, *n* = 26) and anemia (18.5%, *n* = 10). A total of 29 patients (28.7%) experienced at least one serious AE, with leukopenia and nausea/vomiting being the most commonly reported. One patient (5.3%) experienced a serious AE related to anlotinib, which was hand–foot skin reaction. The adverse reactions are listed in Table 3.

**TABLE 3:** Summary of adverse reactions.

	CT (*n* = 49)		TCC (*n* = 19)		ICC (*n* = 33)		Total (*n* = 101)	
	Any grade	Grade ≥ 3	Any grade	Grade ≥ 3	Any grade	Grade ≥ 3	Any grade	Grade ≥ 3
Patients with ≥ 1 AE	47	16 (34%)	19	6 (31.6%)	33	12 (36.4%)	99	34 (34.3%)
Hypertension	0	0	4	0	0	0	4	0
Leukopenia	21	10	11	5	21	11	53	25
Anemia	27	0	14	0	23	0	64	0
Neutropenia	17	5	8	2	25	6	50	13
Increased ALT/AST	7	0	3	0	4	0	14	0
Increased ALP	4	0	0	0	7	0	11	0
Weakness	24	0	9	0	19	0	52	0
Decreased appetite	18	0	8	1	13	0	39	0
Nausea	32	0	13	0	21	0	66	0
Hand–foot syndrome	0	0	4	1	0	0	4	1
Vomiting	12	1	5	0	9	0	26	0
Hypoproteinemia	17	1	14	2	13	0	44	0
Diarrhea	1	0	0	0	0	0	1	0
Immune‐related pneumonia	0	0	0	0	1	0	1	0
Immune‐related hepatitis	0	0	0	0	2	0	2	0

## 4 Discussion

SCLC is considered one of the most aggressive and unfavorable types of lung cancer, often associated with a significantly poor prognosis. The average OS typically ranges from only 2 to 4 months [14], and the median OS rarely exceeds 6–10 months even with intensive treatment [15]. Despite SCLC's high sensitivity to chemotherapy, recurrence rates remain high, exceeding 80% within the first year after initial therapy [6]. When the disease progresses, current treatment modalities lose their effectiveness. Therefore, there is an urgent need for more effective frontline treatment regimens for SCLC patients. While some clinical trials in Japan suggest that irinotecan‐based protocols may offer improved outcomes in ES‐SCLC, the OS advantage remains uncertain [16]. As a result, the search for more effective frontline treatment strategies continues to be a critical area of research.

Immune therapy (IO) has emerged as a significant advancement in cancer treatment, particularly with the introduction of ICIs. SCLC, strongly associated with smoking, exhibits a high tumor mutation burden (TMB) [17], suggesting potential benefits from immunotherapy. PD‐1/PD‐L1 inhibitors are commonly used ICIs that work by restoring T‐cell activity and enhancing the immune response, leading to tumor elimination [18]. Previous studies have shown that combining appropriate chemotherapy regimens with PD‐1/PD‐L1 blockade and optimizing the tumor immune microenvironment can enhance the antitumor immune efficacy of PD‐1/PD‐L1 inhibitors [19]. Numerous studies have confirmed the survival benefits of PD‐1/PD‐L1 inhibitors in patients with ES‐SCLC. For example, preliminary findings from the IMpower133 study demonstrated the survival advantage of atezolizumab combined with cytotoxic agents in ES‐SCLC patients [5]. Compared to the chemotherapy‐only group, the atezolizumab plus chemotherapy group experienced a 2‐month extension in median OS and a 13% higher 1‐year survival rate. The CASPIAN trial, another Phase III study investigating first‐line immunotherapy in ES‐SCLC patients, initially showed a total survival rate of 62.6% and a median OS of 13.0 months for the durvalumab plus chemotherapy group, significantly surpassing the chemotherapy‐only group [6]. Updated results from this study showed an improved total survival rate of 82% and a median OS of 12.9 months for the durvalumab plus chemotherapy group, compared to a median OS of 10.5 months for the chemotherapy‐only group [20]. Other studies are also evaluating the efficacy of PD‐1 inhibitors combined with chemotherapy for ES‐SCLC patients. The KEYNOTE‐604 study [21] aims to compare the efficacy of pembrolizumab plus standard chemotherapy versus standard chemotherapy alone in untreated ES‐SCLC patients. Although preliminary results showed improved PFS with pembrolizumab plus chemotherapy, the final analysis did not reveal a significant improvement in OS. Similarly, another Phase II study [22] reached similar conclusions, demonstrating a statistically significant extension in mPFS with nivolumab plus chemotherapy but no significant difference in the secondary endpoint of OS. Overall, research on immunotherapy for ES‐SCLC has made significant progress and provided new effective treatment options, transforming the frontline treatment approach for ES‐SCLC patients. Domestic PD‐1 agents offer economically feasible treatment alternatives for a wider range of patients.

Tislelizumab is a highly specific and affinity PD‐1 monoclonal antibody designed to minimize binding to FcɣR on macrophages, thus preventing antibody‐dependent phagocytosis and potentially inhibiting T‐cell clearance and PD‐1 therapy resistance mechanisms [8]. Compared to pembrolizumab and nivolumab, tislelizumab has a higher affinity for PD‐1 and demonstrates more stable binding, with in vitro dissociation rates 100 times slower than the former and 50 times slower than the latter [9]. This indicates that tislelizumab can bind more strongly and consistently to PD‐1, enhancing treatment efficacy. These unique characteristics position tislelizumab as a promising candidate for immunotherapy and provide a new treatment option for patients with ES‐SCLC. A Phase III study in ES‐SCLC patients demonstrated that the anti‐PD‐1 antibody serplulimab, when combined with chemotherapy, improved OS compared to chemotherapy alone [23]. Previous research also suggests that tislelizumab may have similar antitumor effects in SCLC and warrants further investigation. A Phase II study indicated that tislelizumab, in combination with etoposide plus platinum, exhibited potent antitumor activity in ES‐SCLC patients, with an mPFS of 6.9 months and a 1‐year OS rate of 76% [10]. Currently, there is limited mature data available regarding tislelizumab combined with chemotherapy as a first‐line treatment for ES‐SCLC. Previous Phase II studies found that introducing tislelizumab to standard etoposide/platinum chemotherapy in untreated ES‐SCLC patients resulted in encouraging PFS and OS, with acceptable tolerability and no new safety signals. This highlights new prospects in the field of immunotherapy for ES‐SCLC. To verify these results, we conducted a retrospective study in the real world, including 33 patients with ES‐SCLC who received tislelizumab in combination with etoposide plus platinum or carboplatin/cisplatin (EP/C) as first‐line treatment. The study findings demonstrated an mPFS of 7.8 months, an overall response rate of 72.7%, and a DCR of 90.9%. These results were statistically superior to those in patients who received chemotherapy alone. Furthermore, the adverse reactions of tislelizumab in combination with EP/C were tolerable, and no unexpected treatment‐related AEs were observed. These findings further support the potential efficacy of tislelizumab as a first‐line treatment for patients with ES‐SCLC. In conclusion, ICIs have shown promising efficacy in patients with ES‐SCLC, thereby changing the frontline treatment approach. The introduction of domestic PD‐1 agents provides economically feasible treatment options for a larger number of patients.

In addition to immunotherapy, the incorporation of antiangiogenic drugs into SCLC treatment is gaining attention. Early research suggests that combining antiangiogenic agents with chemotherapy may yield promising results in first‐line SCLC treatment. Initial studies have shown a correlation between increased microvessel density and high expression of vascular endothelial growth factor (VEGF) protein in SCLC patients, which is associated with a poorer prognosis [24]. This indicates that VEGF may play a crucial role in the development of SCLC, making the inhibition of VEGF a plausible therapeutic strategy. Currently, several antiangiogenic drugs have been evaluated as first‐line treatments for patients with ES‐SCLC, but their clinical efficacy results have been less than satisfactory. For example, in the SALUTE II study, the effectiveness of bevacizumab in combination with standard chemotherapy was assessed for treating ES‐SCLC. The research outcomes revealed an mPFS of 5.5 months in the bevacizumab plus etoposide‐platinum chemotherapy group, surpassing the chemotherapy‐alone group by 1.1 months. However, no statistically significant differences in OS were observed (9.4 months vs. 10.9 months) [25]. In another multicenter, randomized Phase II controlled trial, the addition of rh‐endostatin (recombinant human endostatin) to first‐line chemotherapy did not significantly improve PFS (6.4 months vs. 5.9 months), OS (12.1 months vs. 12.4 months), or complete remission rate (75.4% vs. 66.7%) [26]. Another Phase II study involving 24 S‐SCLC patients showed that the combination of apatinib (a VEGFR‐2 inhibitor) with first‐line chemotherapy extended both PFS (7.8 months vs. 4.9 months) and OS (12.1 months vs. 8.2 months) compared to chemotherapy alone [27]. Given these research findings, further exploration is needed to investigate the potential strategy of combining anlotinib with chemotherapy as a treatment for ES‐SCLC.

Anlotinib is an orally administered TKI that targets VEGF receptors (VEGFRs), platelet‐derived growth factor receptors (PDGFRs), fibroblast growth factor receptors (FGFRs), and c‐kit, resulting in significant antiangiogenic and antitumor effects [28]. The ALTER 1202 study demonstrated the ability of anlotinib to improve the prognosis of patients who have undergone third‐line treatment or beyond for SCLC [13]. Based on these findings, anlotinib was approved by the China Food and Drug Administration (CFDA) in 2019 for the treatment of SCLC in the third‐line setting or beyond. Several small‐sample Phase II clinical trials have shown the superiority of anlotinib in combination with EP/EC chemotherapy as a first‐line treatment for ES‐SCLC. For example, in a clinical trial conducted by Kong et al., 20 ES‐SCLC patients received anlotinib plus platinum‐etoposide chemotherapy as a first‐line treatment, resulting in an mPFS of 10.0 months and a median OS of 15.0 months [29]. Deng et al. reported an mPFS of 9.4 months and a median OS of 13.9 months for anlotinib combined with chemotherapy [30]. In a retrospective study by Zheng et al., 58 ES‐SCLC patients treated with anlotinib plus EP/EC chemotherapy as first‐line therapy showed an mPFS of 6.0 months, a median OS of 10.5 months, an overall response rate (ORR) of 58.6%, and a DCR of 89.7% [31]. This study is aimed at evaluating the efficacy and safety of anlotinib plus platinum chemotherapy as a first‐line treatment for ES‐SCLC patients. A total of 19 patients were enrolled and completed the observation. As of June 30, 2023, the mPFS was 8.0 months, the ORR was 78.9%, and the DCR was 89.5%. These results are consistent with previous research. Anlotinib combined with platinum‐etoposide chemotherapy as a first‐line treatment showed acceptable tolerability, with no unexpected toxicities or treatment‐related deaths observed. Reported nonhematological AEs included known side effects such as fatigue, nausea, hypertension, and hand–foot syndrome, with hypertension being the most common AE. Interestingly, in the study by Song, Xu, and Li, more PFS benefits were observed in patients who developed hypertension or hand–foot syndrome following drug treatment [32]. These findings align with other studies using anlotinib for SCLC, suggesting that anlotinib in combination with platinum‐etoposide does not increase nonhematological toxicity and exhibits acceptable safety. In conclusion, anlotinib combined with platinum chemotherapy as a first‐line treatment for ES‐SCLC shows promising clinical efficacy and acceptable safety. However, further research is required to confirm the advantages and long‐term effects of this combination therapy.

In this retrospective study, we collected clinical data from patients diagnosed with ES‐SCLC and analyzed their impact on patient prognosis. We investigated potential prognostic factors such as age, gender, pathological type, and ECOG performance status. Our statistical analysis identified ECOG performance status as a critical predictor for the prognosis of ES‐SCLC patients. The study results revealed that patients with an ECOG performance status of 0–1 had significantly longer mPFS compared to those with a status of 2 (*p* < 0.05). This finding is consistent with meta‐analyses conducted across different stages of SCLC. The meta‐analyses consistently show that patients with an ECOG performance status of 0–1 have the best prognosis, while those with a score of ≥ 2 have the worst prognosis [33]. A higher ECOG performance status is associated with shorter survival [34].

## 5 Conclusion

Our research findings suggest that targeted therapy combined with chemotherapy and immunotherapy combined with chemotherapy offer superior short‐term and long‐term efficacy compared to traditional chemotherapy in ES‐SCLC patients. Particularly, the combination of immunotherapy and chemotherapy demonstrated the most favorable outcomes in terms of PFS. Additionally, the incidence and types of adverse reactions were similar among the different treatment groups. Therefore, this study provides valuable information for physicians when selecting first‐line treatment options for ES‐SCLC patients, with the goal of enhancing treatment effectiveness and prognosis. However, it is important to note the limitations of this study, including its retrospective design and single‐center data collection. Hence, larger‐scale, multicenter, randomized controlled trials are needed to validate these results. Considering the complexities of ES‐SCLC, future research can explore comprehensive treatment strategies involving the combined applications of chemotherapy, targeted therapy, RT, and immunotherapy. These approaches may further enhance treatment effectiveness and improve patients' survival prognosis.

## Author Contributions

Tiantian Zhang designed the research framework, collected and analyzed data, and drafted the manuscript.

Rui Wang provided research direction and guidance, reviewed and edited the paper, and communicated with journal editors and peers.

Lu Tao assisted in data collection and analysis, participated in discussions and interpretation of research findings, and contributed to the revision and polishing of the manuscript.

Yufo Chen contributed to the research design, assisted in data collection and analysis, and provided expertise and technical support.

Shanshan Zhang assisted in data analysis and interpretation, participated in discussions, and contributed to the writing of specific sections of the paper.

Yang Liu provided experimental equipment and instrument support and participated in data analysis and interpretation of results.

Yumei Li contributed to the research design and data collection and assisted in the revision and proofreading of the manuscript.

## Disclosure

The authors have nothing to report.

## Ethics Statement

As the study is a retrospective study, it did not involve any direct intervention or interaction with patients. Therefore, the study is exempted from obtaining informed consent as it did not pose any risks to the health and interests of the patients.

## Conflicts of Interest

The authors declare no conflicts of interest.

## Data Availability

Data sharing not applicable to this article as no datasets were generated or analyzed during the current study.
